# Painful muscle fibrosis following synthol injections in a bodybuilder: a case report

**DOI:** 10.1186/1752-1947-6-248

**Published:** 2012-08-20

**Authors:** Suleiman Ghandourah, Markus J Hofer, Andreas Kießling, Bilal El-Zayat, Markus Dietmar Schofer

**Affiliations:** 1 Department of Orthopaedics, University Hospital Marburg, Baldingerstrasse, Marburg 35033, Germany; 2 Department of Neuropathology, University Hospital Marburg, Baldingerstrasse, Marburg 35033, Germany; 3 Department of Radiology, University Hospital Marburg, Baldingerstrasse, Marburg 35033, Germany

## Abstract

**Introduction:**

Synthol is a site enhancement oil used by bodybuilders to boost the cosmetic appearance of muscles. Here, we describe the case of a patient with severe side effects following repeated intramuscular injections of synthol in his right biceps muscle.

**Case presentation:**

A 29-year-old Middle Eastern male bodybuilder, following intramuscular injections of synthol five years ago, presented with painful pressure in his right upper arm. On presentation to our clinic, his muscle appeared disfigured. Magnetic resonance imaging revealed scattered cystic fatty lesions in the muscle. The affected part was surgically removed and histopathology showed inflammatory changes with fibrosis and a so-called Swiss cheese pattern.

**Conclusion:**

Synthol injections that are used for the short-term enhancement of muscle appearance by bodybuilders bear the danger of long-term painful muscle fibrosis and disfigurement.

## Introduction

Site enhancement oils were first introduced in 1899 for the purposes of breast augmentation and the filling of wrinkles [1,2]. Synthol, one of the substances used for this purpose, is composed of 85% oil (medium-chain triglycerides), 7.5% lidocaine and 7.5% alcohol. Following injection with synthol, the injected muscle undergoes immediate enlargement. However, this method can also result in muscle deformity [3].

## Case presentation

A 29-year-old Middle Eastern male bodybuilder with a history of prior repeated synthol injections presented at our clinic with ongoing pain and deformity in both upper arms. At the age of 25, our patient had 3mL synthol repeatedly injected by an unlicensed friend into both biceps brachii muscles. Injections were administered four times per week for a total period of four weeks. The total number of injections was 16 injections per biceps muscle. Our patient experienced pain and pressure in the injected muscle directly after each injection. The pain was rated initially as four using a visual analogue scale retrospectively. Despite this, a training session was carried out by our patient after each injection. His perception of pain gradually increased to six out of ten, and after two years our patient ceased training due to the severe pain (rated seven out of ten). Initially, our patient had been able to withstand the pain but, after two years of drug administration, the pain was increasing and not tolerable due to its constant and persistent nature.

**Figure 1:**
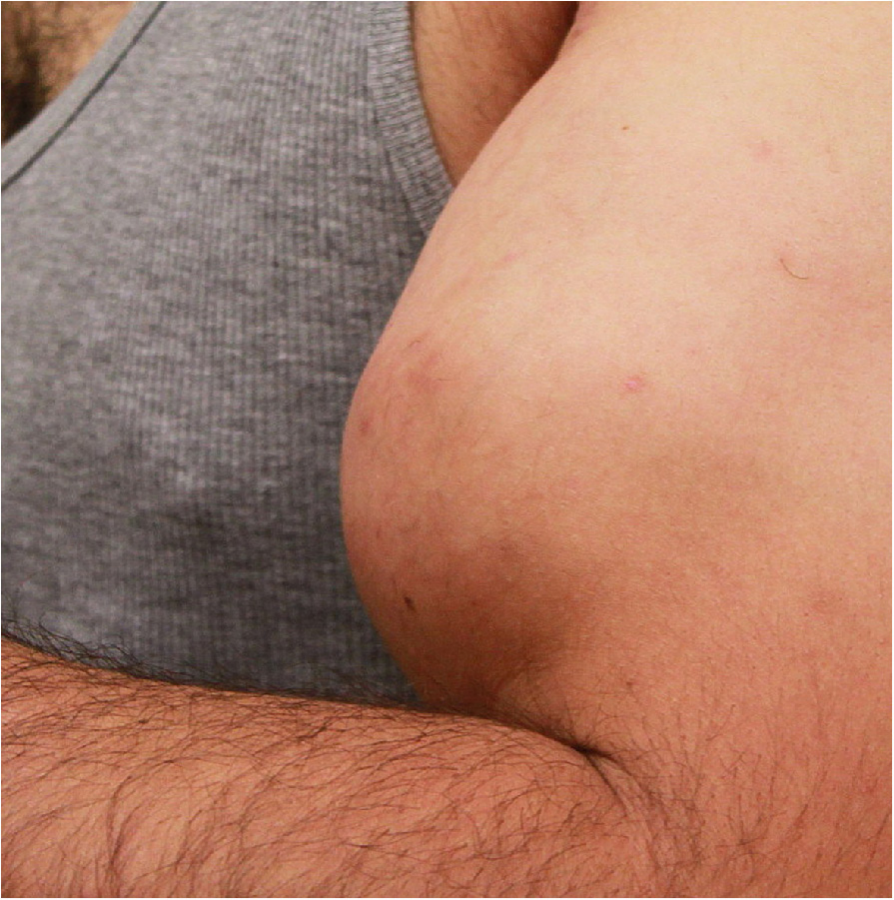
**Preoperative appearance of left biceps muscle.** Right hand not shown due to distinguishing features.

**Figure 2:**
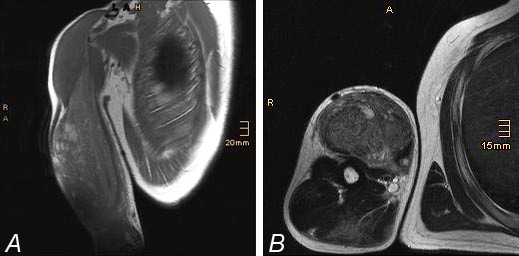
**Magnetic resonance image of swollen biceps muscle after intramuscular injection of synthol in (A) coronal T1-weighted turbo spin echo sequence and (B) transverse T2-weighted turbo spin echo sequence.** Due to the presence of fat in the injected suspension, the total muscle had a hyperintense signal in T1-weighted and T2-weighted images compared with the surrounding healthy muscles. Several cystic lesions are scattered within the muscle with a hyperintense signal. These lesions are oil deposits between the muscle bundles.

**Figure 3:**
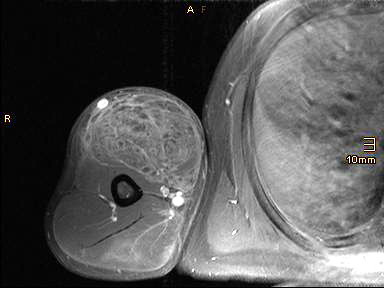
**Gadolinium-enhanced transverse T1-spin echo magnetic resonance image sequence with fat saturation of the right arm.** The inhomogeneous contrast enhancement of the biceps muscle indicates the presence of inflammation.

Upon physical examination, our patient was observed to have rubbery firm hypertrophic and dysmorphic biceps in both arms (Figure 1) with a free range of motion. He complained of a constant painful pressure within his right muscle more than his left one, and muscle deformity. He had several tender points all over his biceps muscle. A diagnosis was made through magnetic resonance imaging (MRI), which revealed a swollen right biceps muscle and cystic lesions scattered throughout the muscle tissue with a hyperintense signal. MRI indicated these lesions to be oil deposits between muscle fibers, termed oleomas (Figure 2). It was concluded that his muscle underwent fibrotic changes in its appearance. In addition, contrast enhancement was inhomogeneous, indicating the presence of inflammation (Figure 3).

For ongoing pain and with no therapeutic alternatives, an open surgical excision of the anterior third of his biceps was carried out through an anterior bicipital approach. Intraoperative findings showed no common muscle tissue left but massive fibrotic tissue similar to scar tissue. Postoperatively, our patient experienced a release of the subjective pain and intracompartmental pressure.

**Figure 4:**
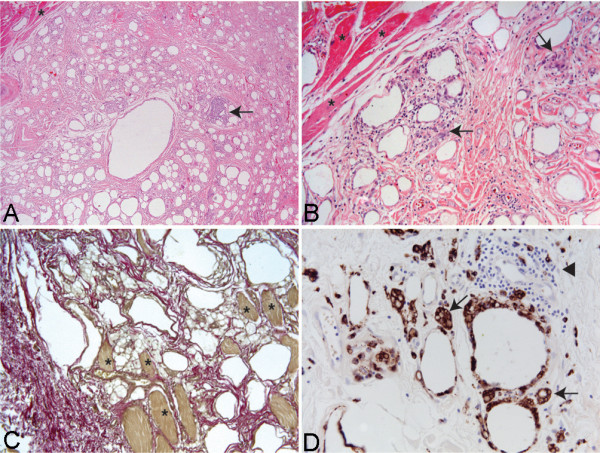
**The overview shows the destroyed muscular architecture and replacement of the muscle by connective tissue.** ( **A**) Residual muscle fibers (asterisks) show pronounced myopathic changes while the connective tissue contains vacuoles with inflammatory infiltrates (arrows). Hematoxylin and eosin stain , ×10 magnification. ( **B**) Higher magnification reveals inflammatory infiltrates in the connective tissue and surrounding the vacuoles. Hematoxylin and eosin stain, ×40 magnification. ( **C**) Elastica van Gieson stain shows remaining muscle fibers (asterisks) with intermingled connective tissue and vacuoles, ×20 magnification. ( **D**) Multinucleated giant cells (arrows) and mononuclear infiltrates (arrowhead, cells negative for CD68) surround vacuoles, ×40 magnification.

The operative specimen, measuring 11.0cm × 5.0cm × 5.0cm, was sent to the Department of Neuropathology for histological examination. The diagnosis was reconfirmed as fibrosis. Sections of the unfixed material revealed a white to yellowish lesion with intermingled small fragments of muscle. Histology showed a predominance of connective tissue with vacuoles (Figure 4A,B,C) and small areas of striated muscle with myopathic changes (Figure 4A,B,C; asterisks). Several necrotic muscle fibers were observed. The connective tissue contained inflammatory infiltrates that were in part diffusely distributed, in part accumulated in foci (Figure 4A,B; arrows). The infiltrates were dominated by CD68+ macrophages (Figure 4D) with numerous multinucleated giant cells (Figure 4D; arrows) and lymphocytes (Figure 4D; arrowhead). Immunohistochemistry identified the lymphocytes as CD4+ and CD8+ T-cells as well as CD20+ B-cells (not shown).

Two weeks postoperatively, our patient was satisfied with the outcome. He was advised to withhold vigorous training for a period of 12 weeks. No complications were reported. After six months, the patient requested the same procedure to be done on his left biceps and surgery was carried out later.

## Discussion

There are not many case reports in the literature that document complications from the use of synthol in bodybuilders. However, there are several reports of complications in patients after injections of paraffin, sesame- and walnut oil [1,4-7]. They all share common histological findings with an inflammatory foreign body reaction, fibrosis and extensive vacuolation [6]. The latter is also known for producing a ‘Swiss cheese’ appearance [5,6], while individual cysts are named according to the injected material, for example, oleoma or paraffinoma [6,7].

Such enhancement oils do not increase muscular strength or performance and are used solely for cosmetic purposes [1]. In the presented case, our patient was under social pressure to use synthol to improve his appearance. However, two years after the injections, the increasing fibrosis and concomitant disfigurement of the muscular appearance caused embarrassment due to his negative body image. Other potential side effects that have been reported include oil embolism, myocardial infarction, cerebral stroke, ulcers and infections [3,6].

## Conclusions

Although the use of synthol by bodybuilders supplies them with the desired short-term effects, the compound progressively destroys the injected muscle. Users are frequently drawn to synthol as it does not share the side effects of androgenic anabolic steroid hormones. However, our case study demonstrates that, despite these perceived advantages, synthol can also have severe and potential life-threatening consequences for its users.

## Consent

Written informed consent was obtained from the patient for publication of this case report and accompanying images. A copy of the written consent is available for review by the Editor-in-Chief of this journal.

## Competing interests

The authors declare that they have no competing interests.

## Authors’ contributions

SG and MDS analyzed and interpreted the patient data regarding synthol intramuscular injections. MDS carried out the surgical intervention on the patient. SG, BEZ and MDS were the main writers of the manuscript. MJH performed the histological examinations of the biceps muscle, AK carried out the radiological examination, and both were major contributors in writing the manuscript. All authors read and approved the final manuscript.
